# Correction: Inferring Tree Causal Models of Cancer Progression with Probability Raising

**DOI:** 10.1371/journal.pone.0115570

**Published:** 2014-12-12

**Authors:** The PLOS ONE Staff

Part of the first author’s last name was incorrectly given as their middle name. The first author’s first name is Loes, and their last name is Olde Loohuis. The correct citation is given below.

Olde Loohuis L, Caravagna G, Graudenzi A, Ramazzotti D, Mauri G, et al. (2014) Inferring Tree Causal Models of Cancer Progression with Probability Raising. PLoS ONE 9(10): e108358. doi:10.1371/journal.pone.0108358
