# Supplementary material for: Inferring Tree Causal Models of Cancer Progression with Probability Raising
Source: PLoS One. 2014 Oct 9;9(10):e108358. doi: 10.1371/journal.pone.0108358 (PMC4191986; doi:10.1371/journal.pone.0108358)
Supplement: File S1 — Contains Proofs, Synthetic Data Generation, and Further Results. (PDF) [file pone.0108358.s001.pdf]

Supporting Information Legends

S1 Proofs

S2 Synthetic data generation

S3 Further results

Supplementary Materials

S1 Proofs

Here the proofs of all the propositions and theorems follow.

Proof of Proposition 1 (Dependency).

Proof. For $\Rightarrow$ write $\mathcal{P}(\overline{a}, b) = \mathcal{P}(b) - \mathcal{P}(a, b)$, then write the PR as

$$\frac{\mathcal{P}(a,b)}{\mathcal{P}(a)} > \frac{\mathcal{P}(b) - \mathcal{P}(a,b)}{1 - \mathcal{P}(a)}$$

and, since $0 < \mathcal{P}(a) < 1$, the proposition follows by simple algebraic arrangements of $\mathcal{P}(a, b) \cdot [1 - \mathcal{P}(a)] > \mathcal{P}(a)\mathcal{P}(b) - \mathcal{P}(a, b) \cdot \mathcal{P}(a)$. The derivations are analogous but in reverse order for the implication $\Leftarrow$. □

Proof of Proposition 2 (Mutual probability raising).

Proof. The proof follows by Property 1 and the subsequent implication:

$$\mathcal{P}(b \mid a) > \mathcal{P}(b \mid \overline{a}) \Leftrightarrow \mathcal{P}(a, b) > \mathcal{P}(a)\mathcal{P}(b) \Leftrightarrow \mathcal{P}(a \mid b) > \mathcal{P}(a \mid \overline{b})\,.$$

□

Proof of Proposition 3 (Probability raising and temporal priority).

Proof. We first prove the forward direction $\Rightarrow$. Let $x = \mathcal{P}(\overline{a}, b)$, $y = \mathcal{P}(a, b)$ and $z = \mathcal{P}(a, \overline{b})$. We have two assumptions we will use later on:

1. $\mathcal{P}(a) > \mathcal{P}(b)$ which implies $\mathcal{P}(\overline{a}, b) < \mathcal{P}(a, \overline{b})$, i.e., $x < z$.
2. $\mathcal{P}(a \mid b) > \mathcal{P}(a \mid \overline{b})$ which, when $0 < x + y < 1$, implies by simple algebraic rearrangements the inequality
$$y[1 - x - y - z] > xz\,. \tag{13}$$

We proceed by rewriting $\mathcal{P}(b \mid a)/\mathcal{P}(b \mid \overline{a}) > \mathcal{P}(a \mid b)/\mathcal{P}(a \mid \overline{b})$ as

$$\frac{\mathcal{P}(a,b)\mathcal{P}(\overline{a})}{\mathcal{P}(\overline{a},b)\mathcal{P}(a)} > \frac{\mathcal{P}(a,b)\mathcal{P}(\overline{b})}{\mathcal{P}(a,\overline{b})\mathcal{P}(b)}$$

which means that

$$\frac{\mathcal{P}(b \mid a)}{\mathcal{P}(b \mid \overline{a})} > \frac{\mathcal{P}(a \mid b)}{\mathcal{P}(a \mid \overline{b})} \iff \frac{\mathcal{P}(\overline{a})}{\mathcal{P}(\overline{a},b)\mathcal{P}(a)} > \frac{\mathcal{P}(\overline{b})}{\mathcal{P}(a,\overline{b})\mathcal{P}(b)} \tag{14}$$

We can rewrite the right-hand side of (14) by using x, y, z where $\mathcal{P}(a) = \mathcal{P}(a, b) + \mathcal{P}(a, \overline{b}) = y + z$ and $\mathcal{P}(b) = \mathcal{P}(a, b) + \mathcal{P}(\overline{a}, b) = x + y$, and then do suitable algebraic manipulations. We have

$$\frac{1 - y - z}{x(y + z)} > \frac{1 - x - y}{z(x + y)} \iff yz - y^2z - xz^2 - yz^2 > xy - x^2y - x^2z - xy^2 \tag{15}$$

when $x(y+z) \neq 0$ and $z(x+y) \neq 0$. To check that the right side of (15) holds we show that

$$(xy - x^2y - x^2z - xy^2) - (yz - y^2z - xz^2 - yz^2) < 0\,.$$

First, we rearrange it as $(x-z)[y - y^2 - xz - y(x+z)] < 0$ to show that

$$(x-z)[y(1-y-x-z) - zx] < 0 \tag{16}$$

is always negative. By observing that, by assumption 1 we have $z > x$ and thus $(x-z) < 0$, and, by equation (13) we have $y(1-y-x-z) - zx > 0$, we derive

$$\frac{\mathcal{P}(b \mid a)}{\mathcal{P}(b \mid \overline{a})} > \frac{\mathcal{P}(a \mid b)}{\mathcal{P}(a \mid \overline{b})}$$

which concludes the $\Rightarrow$ direction.

The other direction $\Leftarrow$ follows immediately by contraposition: assume that $\mathcal{P}(a \mid b) > \mathcal{P}(a \mid \overline{b})$, $\mathcal{P}(b \mid a)/\mathcal{P}(b \mid \overline{a}) > \mathcal{P}(a \mid b)/\mathcal{P}(a \mid \overline{b})$ and $\mathcal{P}(b) \leq \mathcal{P}(a)$. We distinguish two cases:

1. $\mathcal{P}(b) = \mathcal{P}(a)$, then $\mathcal{P}(b \mid a)/\mathcal{P}(b \mid \overline{a}) = \mathcal{P}(a \mid b)/\mathcal{P}(a \mid \overline{b})$.
2. $\mathcal{P}(b) < \mathcal{P}(a)$, then by symmetry $\mathcal{P}(b \mid a) > \mathcal{P}(b \mid \overline{a})$, and by the $\Rightarrow$ direction of the proposition it follows that $\mathcal{P}(b \mid a)/\mathcal{P}(b \mid \overline{a}) < \mathcal{P}(a \mid b)/\mathcal{P}(a \mid \overline{b})$.

In both cases we have a contradiction. This completes the proof. □

Proof of Proposition 4 (Monotonic normalization).

Proof. We prove the forward direction $\Rightarrow$, the converse follows by a similar argument. Let us assume

$$\frac{\mathcal{P}(b \mid a)}{\mathcal{P}(b \mid \overline{a})} > \frac{\mathcal{P}(a \mid b)}{\mathcal{P}(a \mid \overline{b})} \tag{17}$$

then $\mathcal{P}(b \mid a)\mathcal{P}(a \mid \overline{b}) > \mathcal{P}(a \mid b)\mathcal{P}(b \mid \overline{a})$. Now, to show the righthand side of the implication, we will show that

$$\Big[\mathcal{P}(b \mid a) - \mathcal{P}(b \mid \overline{a})\Big]\Big[\mathcal{P}(a \mid b) + \mathcal{P}(a \mid \overline{b})\Big] > \Big[\mathcal{P}(b \mid a) + \mathcal{P}(b \mid \overline{a})\Big]\Big[\mathcal{P}(a \mid b) - \mathcal{P}(a \mid \overline{b})\Big]$$

which reduces to show

$$\mathcal{P}(b \mid a)\mathcal{P}(a \mid \overline{b}) - \mathcal{P}(b \mid \overline{a})\mathcal{P}(a \mid b) > \mathcal{P}(b \mid \overline{a})\mathcal{P}(a \mid b) - \mathcal{P}(b \mid a)\mathcal{P}(a \mid \overline{b})\,.$$

By (17), two equivalent inequalities hold

$$\mathcal{P}(b \mid a)\mathcal{P}(a \mid \overline{b}) - \mathcal{P}(b \mid \overline{a})\mathcal{P}(a \mid b) > 0$$
$$\mathcal{P}(b \mid \overline{a})\mathcal{P}(a \mid b) - \mathcal{P}(b \mid a)\mathcal{P}(a \mid \overline{b}) < 0$$

and hence the implication holds. □

Proof of Proposition 5 (Coherence in dependency and temporal priority).

Proof. We make two assumptions:

1. $\mathcal{P}(b \mid a) > \mathcal{P}(b \mid \overline{a})$ which implies $\alpha_{a \to b} > 0$.
2. $\mathcal{P}(a, b) > \mathcal{P}(a)\mathcal{P}(b)$ which implies $\beta_{a \to b} > 0$.

The proof for dependency follows by Proposition 1 and its implication:

$$\mathcal{P}(b \mid a) > \mathcal{P}(b \mid \overline{a}) \Leftrightarrow \mathcal{P}(a,b) > \mathcal{P}(a)\mathcal{P}(b) \Leftrightarrow \alpha_{a\to b} > 0 \Leftrightarrow \beta_{a\to b} > 0.$$

Moreover, being β symmetric by definition, the proof for temporal priority follows directly by Proposition 2 $\square$

The properties outlined so far are sketched informally in the following diagram.

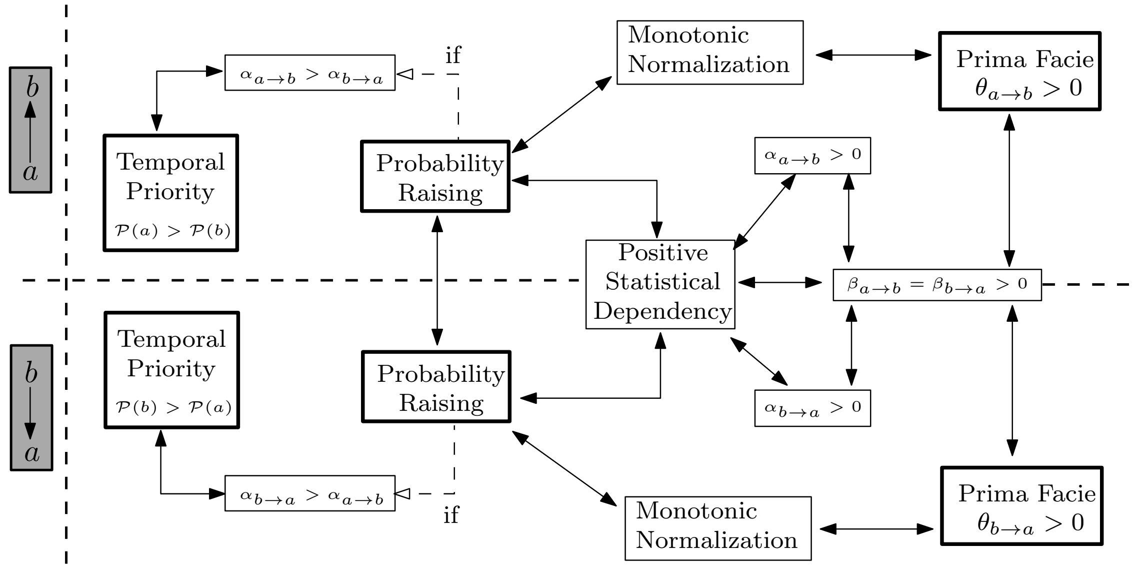

Proof of Theorem 1 (Independent progressions).

Proof. For any $a_i \in G^*$ it holds that

$$\mathcal{P}(a_i) > \mathcal{P}(b) \qquad m_{a_i\to b} > 0$$

being prima facie, also $a^* \to b$ is the edge selected by CAPRESE being the $\max\{\cdot\}$ over G^*. Thus, CAPRESE selects $\diamond \to b$ instead of $a^* \to b$ if, for any a_i, it holds

$$\frac{1}{1+\mathcal{P}(b)} > \frac{\mathcal{P}(a_i)}{\mathcal{P}(a_i)+\mathcal{P}(b)}\frac{\mathcal{P}(a_i,b)}{\mathcal{P}(a_i)\mathcal{P}(b)}\,.$$

With some algebraic manipulations we rewrite this as

$$\mathcal{P}(a_i)\mathcal{P}(b) + \mathcal{P}(b)^2 > \mathcal{P}(a_i,b)(1+\mathcal{P}(b))\,,$$

which gives the inequality in the theorem statement. $\square$

Proof of Theorem 2 (Algorithm correctness).

Proof. It is clear that CAPRESE does not create disconnected components since, to each node in G, a unique parent is attached (either from G or $\diamond$). For the same reason, no transitive connections can appear.

The absence of cycles results from Properties 3, 4 and 5. Indeed, suppose for contradiction that there is a cycle $(a_1,a_2),(a_2,a_3),\ldots,(a_n,a_1)$ in E, then by the three propositions we have

$$\mathcal{P}(a_1) > \mathcal{P}(a_2) > \ldots > \mathcal{P}(a_n) > \mathcal{P}(a_1)$$

which is a contradiction. $\square$

Proof of Theorem 3 (Asymptotic convergence).

Proof. For any $u \in G$, when $s \to \infty$ the *observed probability* $\mathcal{P}(u)$ (evaluated from D) is equivalent to the product of the probabilities (in T) obtained by traversing the forest from the root $\diamond$ to u (Definition 1). Thus, $\mathcal{P}(u) \in (0,1)$ since the traversal probabilities are in $(0,1)$ too, hence all events are distinguishable and Algorithm 1 reconstructs a tree with the same events set G of T.

We now observe that the distribution induced by T (Definition 1) respects a *single-cause prima facie topology* where to each event is assigned *at most* a single cause. In other words, Definition 2 holds for any edge $(u,v) \in E$:

- by the event-persistence property usually assumed in cancer (fixating mutations are present in the progeny of a clone) the occurring times satisfy $t_u < t_v$ which, in a frequentist sense, implies $\mathcal{P}(u) > \mathcal{P}(v)$;
- it holds by construction (Definition 1) that $\mathcal{P}(v,u) = \mathcal{P}(v)$, thus $\mathcal{P}(v \mid u) = \mathcal{P}(v)/\mathcal{P}(u)$ which is strictly positive since $\mathcal{P}(v)$ and $\mathcal{P}(u)$ are, and that $\mathcal{P}(v,\overline{u}) = 0$, thus $\mathcal{P}(v \mid \overline{u}) = 0$.

To correctly reconstruct T we rely on the fact that our score $m_{u \to v}$ is consistent with the prima facie probabilistic causation because of:

- Proposition 3, which states that PR (embedded as $\alpha_{u \to v}$ in m) subsumes a good temporal priority model of occurring times, as stated above;
- Proposition 4 and 5 which ensure the monotonicity and sign coherency among $\alpha_{u \to v}$ and $\beta_{u \to v}$ in m.

Thus, m is consistent with a single-cause prima facie topology. We now show that Algorithm 1 reconstructs correctly a generic edge in E, and hence also T.

Consider an event $v \in G$ and edge $(u,v) \in E$. The set of its "candidate" parent events is $G \setminus \{v\}$, we partition it in three disjoint sets $\mathcal{G}$, $\mathcal{S}$ and $\mathcal{N}$:

- $\mathcal{G}$*, genuine:* all the backward-reachable events, in $G \setminus \{v\}$, from v;
- $\mathcal{S}$*, spurious (or ambiguous):* all the events (but v) in the sub-forest which includes the path from $\diamond$ to v, which are not in $\mathcal{G}$;
- $\mathcal{N}$*, non prima facie:* all other events, i.e., $G \setminus (\{v,\} \cup \mathcal{S} \cup \mathcal{G})$;

Notice that $G = \{v\} \cup \mathcal{G} \cup \mathcal{S} \cup \mathcal{N}$, that $u \in \mathcal{G}$ and that all the effects of v are non prima facie to v because of the temporal priority, as shown below.

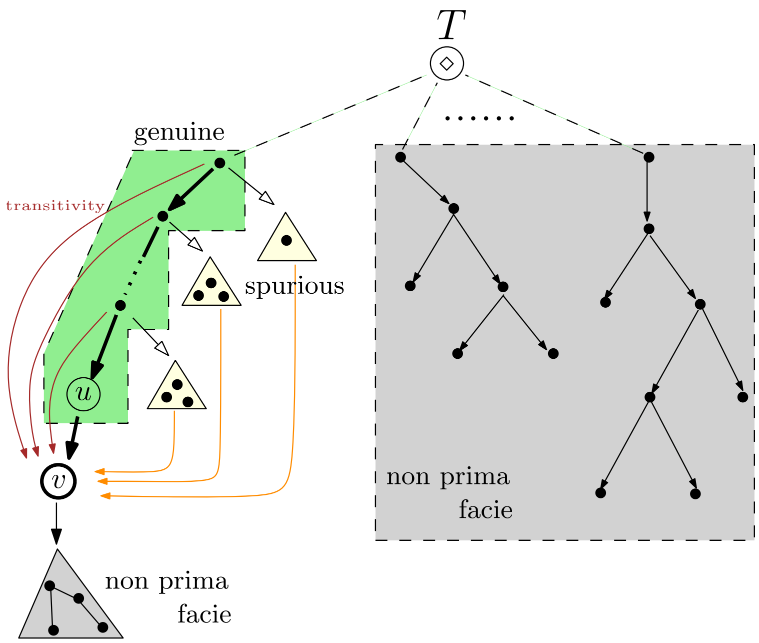

This way of partitioning events according to the structure of T subsumes a equivalent partitioning based on the score $\alpha \in [0,1]$, which we use to prove correctness of our algorithm: for any $x \in G$ it holds that $\alpha_{x \to v} = 1$ if $x \in \mathcal{G}$, $0 < \alpha_{x \to v} < 1$ if $x \in \mathcal{S}$ and $\alpha_{x \to v} < 0$ if $x \in \mathcal{N}$.

We now show that CAPRESE correctly selects $u \in \mathcal{G}$:

- a non prima facie event $x \in \mathcal{N}$ either satisfies (Proposition 1)

$$\mathcal{P}(x, v) \le \mathcal{P}(x)\mathcal{P}(v)$$

  which means that $\alpha_{x \to v} < 0$, $\beta_{x \to v} < 0$ (Proposition 5) and thus $m_{x \to v} < 0$, or it is a descendant of v, which means that $\mathcal{P}(x) < \mathcal{P}(v)$. By construction, CAPRESE considers as candidate parents of v only not descendant events with positive score (see step 3);

- a spurious event $x \in \mathcal{S}$ is prima facie to v but $\alpha_{x \to v} < 1$ since:
  - $\mathcal{P}(x)\mathcal{P}(v) < \mathcal{P}(v, x) < \mathcal{P}(v)$, otherwise x would be backward reachable from x and thus in $\mathcal{G}$;
  - $0 < \mathcal{P}(v, \overline{x}) = \mathcal{P}(v) - \mathcal{P}(v, x)$ which means that $\mathcal{P}(v, \overline{x}) < \mathcal{P}(\overline{x})\mathcal{P}(v)$;
  - by all of the above $\mathcal{P}(v \mid \overline{x}) > 0$ which implies that $\alpha_{x \to v} < 1$.

  Recall now that $\lambda \to 0$, which means that $m_{x \to v} \approx \alpha_{x \to v} < 1$. CAPRESE will thus not select any of these events as cause of v if there exist an event with $m_{x \to v} = 1$, which is actually the case with genuine causes;

- genuine causes are the real cause of v, u, plus *all* the transitive backward-reachable events. Any x of these has maximum score $\alpha_{x \to v} = 1$ since:
  - $\mathcal{P}(x)\mathcal{P}(v) < \mathcal{P}(v, x) = \mathcal{P}(v)$ and $0 = \mathcal{P}(v, \overline{x})$;
  - by the above $\mathcal{P}(v \mid \overline{x}) = 0$ which implies $\alpha_{x \to v} = 1$.

Thus, CAPRESE will pick an event from $\mathcal{G}$, and not from $\mathcal{S}$. We need to show that u is the event with maximum score.

Enumerate the events in $\mathcal{G}$ as g_1 (which is u), ..., g_k in a way that

$$\mathcal{P}(g_1) < \ldots < \mathcal{P}(g_k)$$

and recall that this is a *total ordering* induced by the temporal priority, and that this is consistent with coefficient β, which means that

$$\beta_{g_1 \to v} > \ldots > \beta_{g_k \to v} \,.$$

Thus, in the limit $\lambda \to 0$

$$\max\{m_{g_i \to v} \mid g_i \in \mathcal{G}\} \overset{\lambda \to 0}{\approx} 1 + \max\{\beta_{g_i \to v} \mid g_i \in \mathcal{G}\} \approx 1 + \beta_{g_1 \to v} \approx m_{u \to v}$$

is the event closer in time to v, with respect to β. This event, namely u, is chosen by the algorithm as the real cause of v.

Finally, we show that the last step of the algorithm (the independent progression filter, step 4), does not invalidate the edge (u, v). In fact, the algorithm would replace such an edge with $(\diamond, v)$ if, for all nodes x backward-reachable from v (i.e., those in $\mathcal{G} \cup \mathcal{S}$) it was

$$\frac{1}{1 + \mathcal{P}(v)} > \frac{\mathcal{P}(x)}{\mathcal{P}(x) + \mathcal{P}(v)} \frac{\mathcal{P}(x, v)}{\mathcal{P}(x)\mathcal{P}(v)} \,.$$

It suffices thus to show that the above inequality is violated just by one of the backward-reachable nodes. We pick just $u \in \mathcal{G}$ and note that

$$\frac{\mathcal{P}(u)}{\mathcal{P}(u) + \mathcal{P}(v)} \frac{\mathcal{P}(u, v)}{\mathcal{P}(u)\mathcal{P}(v)} = \frac{\mathcal{P}(u \mid v)}{\mathcal{P}(u) + \mathcal{P}(v)} \,.$$

Also, we have that $\mathcal{P}(u) < 1$, $\mathcal{P}(v) < 1$ and, by construction, $\mathcal{P}(u \mid v) = 1$ because all the instances of v are co-occurring with those of u (but not the converse). Thus, inequality

$$\frac{1}{1 + \mathcal{P}(v)} < \frac{1}{\mathcal{P}(u) + \mathcal{P}(v)} \,,$$

is always true and ensures that edge (u, v) is maintained, which concludes the proof. □

Proof of Corollary 1 (Uniform noise).

Proof. As shown in [22], the uniform rates ϵ_+ and ϵ_- affect the observed probabilities as follows

$$\mathcal{P}(i)^* = \mathcal{P}(i)(1 - \epsilon_-) + (1 - \mathcal{P}(i))\epsilon_+ \tag{18}$$

$$\mathcal{P}(i, j)^* = \mathcal{P}(i, j)(1 - \epsilon_-)^2 + [\pi_{ij} - \mathcal{P}(i, j)](1 - \epsilon_-)\epsilon_+ + (1 - \pi_{ij})\epsilon_+^2 \,, \tag{19}$$

where $\pi_{ij} = \mathcal{P}(i) + \mathcal{P}(j) - \mathcal{P}(i, j)$. It is important to note (Lemma 1, [22]) that

$$\mathcal{P}(i) > \mathcal{P}(j) \implies \mathcal{P}(i)^* > \mathcal{P}(j)^* \,,$$

namely uniform noise is still implying temporal priority. Because of this, and since the raw estimate α is monotonic relative to temporal priority, all the derivations for Theorem 3 are still valid in this context, and the algorithm selects the correct genuine cause for each effect.

To guarantee that no valid connection is broken by the independent progressions filter, we again rely on Szabo's result (Reconstruction Theorem 1, [22]). In particular, for any correctly selected edge (u, v) in our algorithm, since we implement Desper's filter (or, analogously, Szabo's) for independent progressions we do not mistake by deleting (u, v) unless also their algorithms do. Since this is not the case when $\epsilon_+ < \sqrt{p_{\min}}(1 - \epsilon_+ - \epsilon_-)$ the proof is concluded. □

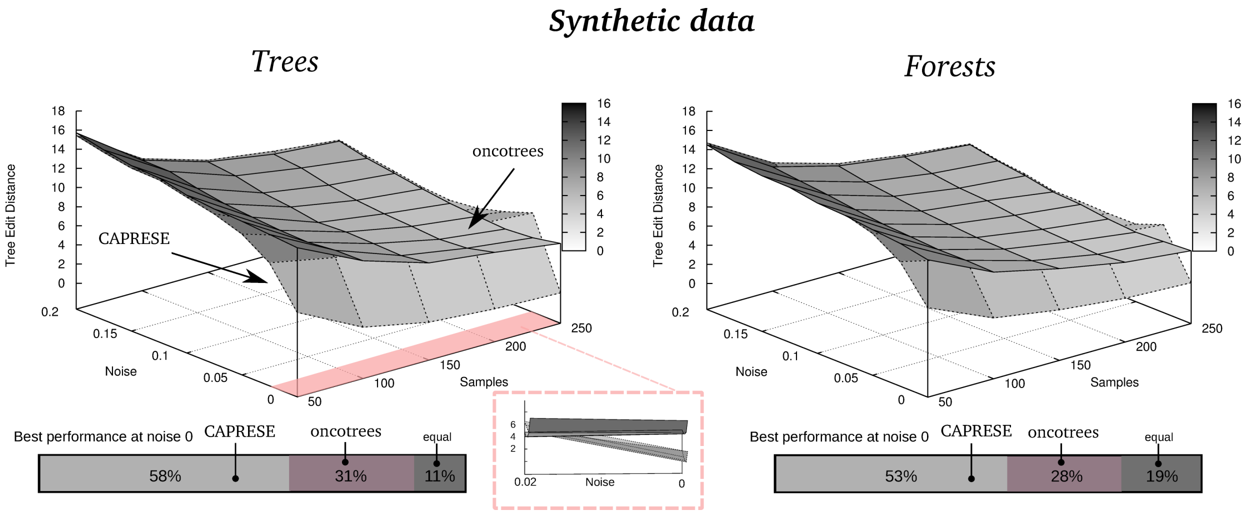

Figure S1: **Reconstruction with noisy synthetic data and $\lambda \to 0$.** The settings of the experiments are the same as those used in Figure 6, but in this case the estimator is shrank by $\lambda \to 0$, i.e., $\lambda = 0.01$. In the magnified image one can sees that the performance of CAPRESE converges to Desper's one already for $\nu \approx 0.01$, hence largely faster than in the case of $\lambda \approx 1/2$ (Fig. 6).

S2 Synthetic data generation

A set of random trees is generated to prepare synthetic tests. Let n be the number of considered events and let $p_{\min} = 0.05 = 1 - p_{\max}$, a *single tree* with maximum depth $\log(n)$ is generated as follows:

pick an event $r \in G$ as the tree root;
assign to each event but r an integer value in $[2, \log(n)]$ representing its depth in the tree, ensure that for each level there is at least one event (0 is reserved for $\diamond$, 1 for r);
for all events $e \neq r$ **do**
  let l be the level assigned to e;
  assign a father to e selecting an event among those at which level $l-1$ was assigned;
  add the selected pair to the set of edges E;
end for
for all edges $(i, j) \in E$ **do**
  assign $\alpha((i, j))$ a random value in $[p_{\min}, p_{\max}]$;
end for
return the generated tree;

When a forest is to be generated, we repeat the above algorithm to create its constituent trees. These trees (or forests), in turn, are used to sample the input matrix for the reconstruction algorithms, with the parameters described in the main text.

S3 Further results

We show here the results of the experiments discussed but not presented in the main text.

Reconstruction of noisy synthetic data with $\lambda \to 0$. Although we know that $\lambda \to 0$ is not the optimal value of the shrinkage-like coefficient for noisy data, we show in Figure S1 the analogue of Figure 6 when the estimator is shrank by $\lambda \to 0$, i.e., $\lambda = 0.01$. When compared to Figure 6 it is clear that a best performance of CAPRESE is obtained with $\lambda \approx 1/2$, as suggested by Figure 2.

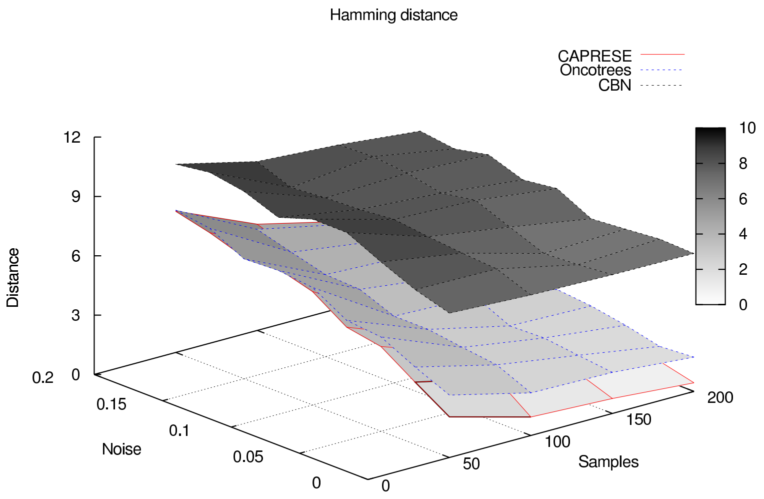

Figure S2: **Reconstruction with CAPRESE compared to oncotrees and h-CBN with noisy synthetic data.** Performance of CAPRESE compared to oncotrees and h-CBNs as a function of the number of samples and noise ν. The λ parameter used for CAPRESE is 1/2, and the reconstructed topologies contain 10 nodes each.

Comparison with hidden Conjunctive Bayesian Networks, h-CBNs. We here compare the performance of CAPRESE to hidden Conjunctive Bayesian Networks (h-CBN)[15], as well as to oncotrees. The settings of the experiment are slightly different from those of the previous analyses: we used 100 distinct random trees of 10 events each. We ranged the number of samples available for reconstruction from 50 to 200, with a step size of 50. The settings used for running h-CBNs are relatively standard settings: we allowed for 50 annealing iterations with initial temperature equal to 1. Since h-CBNs reconstruct DAGs, it is not possible to quantify its performance using Tree Edit Distance, as we did in the comparison with oncotrees. Instead, we here adopt Hamming Distance (computed on the connection adjacency matrix), as a closely related and computationally feasible alternative for measuring performance [33].

The results of the experiment can be found in Figure S2, and show that CAPRESE clearly outperforms h-CBNs. In particular, it is possible to notice that, for all the analyzed values of noise and sample sizes, both CAPRESE and oncotrees display a (average) Hamming Distance between the reconstructed model and the original tree topology that is significantly lower than h-CBNs, with the largest differences observed in the noise-free case. This result would point at a much faster convergence of CAPRESE with respect to the number of samples, also in presence of moderate levels of noise.

A few remarks are warranted about this experiment. First, in contrast to the comparison with oncotrees, we ran each experiment exactly once rather than averaging the results over 10 repetitions, and on relatively smaller trees. These limitations are a consequence of the extremely high time complexity of the simulated annealing step of h-CBNs. However, the comparison between CAPRESE and h-CBNs shows a so large difference in the performance that we do not expect this to be have significant impact. Second, the results obtained by h-CBNs are perhaps worse than expected based on results in the absence of noise presented in [24], which were however based on a unique tree topology. Yet, this outcome may have been potentially influenced by either the estimation procedure of the noise parameter in h-CBN, the adopted annealing procedure or by the used number of iterations. In future work we plan to extend our algorithm to extract more general topologies and to compare both methods in a greater detail.

Inference of models with multiple conjunctive parents. CAPRESE is specifically tailored to reconstruct models with independent progressions and a unique cause for each event (i.e., trees or forests), while other approaches such as CBNs can reconstruct models where multiple conjunctive parents co-occur to cause

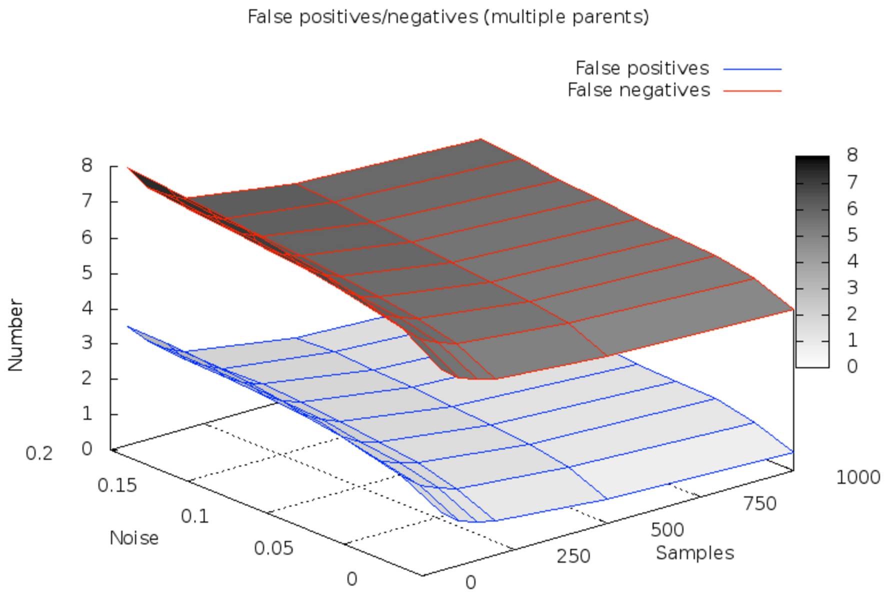

Figure S3: **Performance of CAPRESE to reconstruct models with conjunctive parents and noisy data.** Performance of CAPRESE measured in terms of the number of *false positives/negatives* in the reconstructed model, when data are generated from directed acyclic graphs with 10 nodes and where each event is caused by at most 3 *conjunctive events* (randomly assigned). The λ parameter is set to $1/2$.

an effect (i.e., $a \wedge b$ cause c). It is thus reasonable to use such conjunctive approaches to infer more complex model, in spite of CAPRESE.

However, it is interesting to asses CAPRESE's performance when (synthetic) data are sampled from a model with multiple parents and noise. By sampling input data from random *directed acyclic graphs* with 10 nodes and where each event is caused by at most 3 conjunctive events (randomly assigned), we assess the number of *false positives* and *false negatives* retrieved in the model reconstructed with CAPRESE. We show the results in Figure S3. Our results indicate that for increasing sample size, the number of false positives approaches 0. Thus, for sufficiently large number of samples, all the causal claims returned by CAPRESE are true. In addition, the number of false negatives is always higher and proportional to the connectivity of the target model. This is to be expected since CAPRESE assigns at most one parent (the cause) to every node.
